# Adult attachment and birth experience: importance of a secure base and safe haven during childbirth

**DOI:** 10.1080/02646838.2018.1509303

**Published:** 2018-09-29

**Authors:** Samantha Reisz, Jessica Brennan, Deborah Jacobvitz, Carol George

**Affiliations:** a Department of Public Health and Primary Care, University of Cambridge, Cambridge, United Kingdom of Great Britain and Northern Ireland; b Human Development and Family Sciences, University of Texas at Austin, Austin, TX, USA; c Department of Psychological and Brain Sciences, University of Delaware, Newark, DE, USA; d Family and Consumer Sciences, Modesto Junior College, Modesto, CA, USA; e Human Development & Family Sciences, University of Texas at Austin College of Natural Sciences, Austin, TX, USA; f Psychology, Mills College, Oakland, CA, USA

**Keywords:** Childbirth, attachment, caesarean, parity, mother(s)

## Abstract

**Objective**: Examine connections between mothers’ adult attachment and subjective birth experience in the context of parity and mode of delivery.

**Background**: Research has established a clear connection between adult attachment and birth experience. This study extended previous research with an in-depth self-report attachment measure examining different dimensions of mothers’ attachment representations and their relation to subjective birth experience.  Interactions between mode of delivery and parity were also considered. Method: Participants were 257 mothers who gave birth 4 days to 12 months prior to the study. Mothers’ mean age was 30.5 years, 61% primiparas, and 26% delivered by caesarean.  Participants completed an online survey with the Birth Experience Questionnaire, the Reciprocal Attachment Questionnaire, and demographic information.

**Results**: Hierarchical moderated regression analyses showed direct effects from adult attachment dimensions to mothers’ subjective birth experiences, specifically perceived availability, feared loss, separation protest, angry withdrawal, and compulsive careseeking. Interactions emerged for parity and/or mode of delivery for overall subjective birth experience, perceived control, perceived social support, and satisfaction.

**Conclusion**: Adult attachment representations related to subjective birth experience, indicating that attachment figures serve as secure bases and safe havens for mothers during childbirth. These results have implications for practitioners and provide direction for future research.

## Introduction

Childbirth is a profound experience for women that can influence their relationship with their new babies (Durik, Hyde, & Clark, 2000), confidence in themselves as mothers (Reisz, Jacobvitz, & George, 2015) and their mental health (Olde, van der Hart, Kleber, & Van Son, 2006). The potential for birth to influence maternal outcomes makes it vital to understand what factors are related to how women experience birth. Mothers’ attachment has been connected to birth experience in a small but growing number of studies (Ayers et al., 2014; Quinn, Spiby, & Slade, 2015; Wilson, Rholes, Simpson, & Tran, 2007). Attachment is a social–emotional bond between two people that endures across time and situations (Bowlby, 1969/1982). The main function of attachment in adulthood is to help adults maintain a sense of safety and security (West & Sheldon-Keller, 1992). The primary person that one turns to for this support is termed the *attachment figure*. Based on repeated experiences with attachment figures, individuals develop and carry forward expectations about the self and others in regard to whether others can be counted on to provide support when they are distressed (Bowlby, 1969/1982). Childbirth is reported by most women as distressing, in part due to the intensity of the pain (Wolf, 2009). This distress may activate mothers’ attachment needs, motivating them to seek comforting from a significant other. Hence, it is anticipated that mothers’ perceptions of the birth experience will be related to their ability to reach out to their partners when needed and their sense that their partners will be available, responsive and not abandon them while vulnerable during labour and delivery.

Attachment and birth have only recently received empirical attention; this research has demonstrated clear connections between birth experience and romantic attachment style (Ayers et al., 2014; Quinn et al., 2015; Wilson et al., 2007). Wilson et al. (2007) found that first-time mothers with anxious attachment (e.g. seeking frequent closeness) perceived lower levels of support than mothers with secure or avoidant attachment styles. Quinn et al. (2015) found that higher levels of attachment anxiety corresponded to higher levels of perceived pain. Women with high attachment avoidance (e.g. dismissing the importance of attachment) reported feeling less respected by birthing staff than women high in security or anxiety. Overall, the dimensions associated with compromised security (i.e. anxiety or avoidance) were associated with higher reported levels of acute traumatic stress symptoms and hyperarousal. Ayers and colleagues (2014) reported similar connections between attachment insecurity and posttraumatic stress symptoms. They found a significant positive association between avoidant attachment style and posttraumatic stress symptoms following childbirth, particularly if the women delivered by caesarean section. When taken together, these studies meaningfully link attachment and subjective birth experience, including the potential for the mode of delivery and parity to relate to their experience. Further, the role of adult attachment in the context of birth fits well into the biopsychosocial model of childbirth (Saxbe, 2017), allowing more nuanced understanding of how adult attachment relates to women’s experiences of birth.

Mode of delivery affects mothers’ experience of birth (Carquillat, Boulvain, & Guittier, 2016). Women who delivered by caesarean section reported consistently more negative experiences than those who delivered vaginally (DiMatteo et al., 1996; Fenaroli, Saita, Molgora, & Accordini, 2016; Kjerulff & Brubaker, 2017; Lobel & DeLuca, 2007; Maclean, McDermott, & May, 2000). Caesarean section is a major surgical procedure, and many women expressed concern that the emotional impact of surgical delivery was not adequately addressed during their birth preparations (Murphy, Pope, Frost, & Liebling, 2003). Indeed, feelings of being in control can be a critical factor for mothers deciding whether to attempt a vaginal birth after caesarean (Konheim-Kalkstein, Kirk, Berish, & Galotti, 2017). Quinn et al. (2015) found that subjective birth experience mediated the links between mode of delivery and mothers’ perceptions of their babies as well as their confidence in themselves as mothers. This finding highlights the importance of examining maternal *subjective* experience in conjunction with actual events. Further, caesareans can be necessitated by threats to the health of the mother or baby, which may be particularly distressing and motivate mothers to seek care from their attachment figure. The present study examined interactions between mode of delivery and attachment to determine whether relations between attachment and birth experience varied with delivery mode.

Important factors for women’s birth experiences include perceptions of control (Ford, Ayers, & Wright, 2009; Green & Baston, 2003), social support (Sigurdardottir et al., 2017; Wilson et al., 2007), accuracy of expectations (Murphy et al., 2003; Waldenstrom, 1999) and satisfaction with the overall birth (Green, Coupland, & Kitzinger, 1998). Women who felt in control during labour and delivery, knew what to expect and felt supported by those around them reported better birth experiences than women who were unsure of what was happening, felt unsupported and out of control (Reisz et al., 2015). Parity was related to birth experience in multiple studies, with multiparas reporting more accurate expectations, which fostered feelings of being in control during childbirth (Green et al., 1998; Lobel & DeLuca, 2007; Skari et al., 2002; Waldenstrom, 1999). In addition, primiparas are making the transition to motherhood and may have different experiences and needs than multiparas whose experiences lay the foundation of what to expect from birth as well as parenthood. The attachment needs of both primiparas and multiparas are likely to be activated during labour and delivery, but mothers may respond differently depending on whether they have experienced childbirth before. Thus, the present study examined interactions between parity and attachment to explore whether attachment representation would relate to birth experience based on parity.

This study strengthens the understanding of the connections between birth and attachment representation by examining a range of adult attachment dimensions as they relate to specific aspects of the subjective birth experience. It was expected (a) that mothers’ adult attachment would relate to their subjective birth experience, and that relations between attachment and birth experience would differ based on (b) parity and (c) delivery mode.

## Method

### Participants

Participants were 257 mothers living in the USA who gave birth within 12 months of the study. Infant age ranged from 4 days to 12 months (*M *= 6.8, *SD *= 3.53) with approximately equal numbers of girls and boys (*n *= 131 girls, 51%). Mothers’ ages ranged from 16 to 45 years (*M *= 30.5, *SD *= 5.58). All participants had healthy, singleton births; no infants were admitted to the neonatal intensive care unit. Participant descriptive information can be seen in Table A1.

### Measures

**Birth Experience Questionnaire** (BEQ). The BEQ was adapted from the Green et al. (1998) postpartum questionnaire to assess mothers’ subjective birth experience specifically. The BEQ contains 25 items rated using a seven-point Likert-type scale (1 = *not at all* to 7 = *completely*). There are four subscales associated with factors important to the subjective birth experience: accuracy of expectations (e.g. ‘I feel that I received sufficient information about my mode of delivery and what recovery would be like’, α = .60), perceived control (e.g. ‘I felt that I was in control of what the medical staff did to me’, α = .70), social support (e.g. ‘I feel that my companion(s) was aware of my needs during childbirth’, α = .75) and satisfaction with the birth (e.g. ‘I feel satisfied about the way I delivered’, α = .83). Subscale summary scores are the mean of the subscale items and the BEQ overall summary score was calculated as the mean of the subscales (α = .76). This study examined both mothers’ overall birth experience as well as the specific subscales.

**Reciprocal Attachment Questionnaire** (RAQ; West, Sheldon, & Reiffer, 1987). The RAQ is a representational measure that assesses how individuals perceive their primary adult attachment relationship as it relates to the *functional goal* of attachment security (i.e. proximity to the attachment figure). The questionnaire has 43 items that are rated using a five-point rating scale (1 = *strongly disagree* to 5 = *strongly agree*). The measure assesses five attachment goal-related dimensions: proximity-seeking (e.g. ‘I feel lost if I’m upset and my attachment figure is not around’, α = .63), separation protest (e.g. ‘I resent it when my attachment figure spends time away from me’, α = .62), feared loss (e.g. ‘I would feel helpless without my attachment figure’, α = .83), perceived availability (e.g. ‘I am confident that my attachment figure will try to understand my fears’, α = .82) and use of attachment figure (e.g. ‘I turn to my attachment figure for many things, including comfort and reassurance’, α = .72). Four additional dimensions assess insecure attachment-based personality clusters: compulsive care-giving (e.g. ‘I can’t get on with my work if my attachment figure has a problem’, α = .56), compulsive care-seeking (e.g. ‘I wish I could be a child again and be taken care of by my attachment figure’, α = .68), compulsive self-reliance (e.g. ‘I feel it is best not to depend on my attachment figure’, α = .79) and angry withdrawal (e.g. ‘My attachment figure only seems to notice me when I’m angry’, α = .87). Traditionally, alpha levels are considered acceptable above .60 for this number of items (Cortina, 1993; Loewenthal, 2004). For the one scale of compulsive care-giving with α = .56, it was considered preferable to keep the original and theoretically established scale in the interest of being consistent with prior research using this measure, rather than adjust the scale items for this individual study.

The RAQ was used because it is an in-depth view of adult attachment that measures attachment with multiple dimensions, whereas previous studies have measured attachment using only two dimensions (i.e. anxiety, avoidance). The RAQ dimensions reduce the tendency to view individuals in the context of broad categories and permits evaluating fine-grained aspects of mothers’ attachment representations, following Bowlby (1973). RAQ validity has been established in relation to other adult attachment measures (Ravitz, Maunder, Hunter, Sthankiya, & Lancee, 2010; West, Spreng, Casares-Knight, Rose, & Leiper, 1998) and is reliable in clinical and non-clinical populations (Calabrese, Farber & Weston, 2005; West & Sheldon, 1988; West et al., 1987; West & Sheldon-Keller, 1992).

## Demographics

**Parity** was measured by asking mothers to indicate whether they had previous children and the ages of their other children.

**Income** was assessed by asking mothers to indicate their household income range in USD: Under $10,000, $10,000–$25,000, $25,000–$50,000, $50,000–$75,000, $75,000–$100,000, or over $100,000.

## Procedure

Mothers were recruited using postcard advertisements with the study web link and web announcements posted on parent-oriented websites, such as parenting blogs. Postcards were distributed in toy stores and community centres in major US urban areas. The first page of the survey described the research purpose, terms of participation and required participant consent to continue. Mothers could end the survey at any time. Participants did not receive any compensation for their participation in this study. Data were collected as part of a master’s thesis project, so as many people as possible were recruited in the time frame allotted. The final sample comprised the 257 mothers who completed all of the relevant measures, which is large enough for us to detect at least medium effect sizes for the relationships examined (Cohen, 1992; VanVoorhis & Morgan, 2007).

### Data analysis

A series of hierarchical moderated regression analyses were conducted to examine relations between attachment and subjective birth experience and interactions with parity and delivery mode. Parity and delivery mode were dummy coded such that primiparous = 0 and multiparous = 1, and vaginal = 0 and caesarean = 1. Infant age was included as a control to account for time since delivery. Controls were entered in the first step of each regression. Parity and mode of delivery were entered in step 2. RAQ dimensions were entered in step 3. Interaction terms were created by multiplying the grand centred mean for each attachment dimension by those of parity and mode of delivery and were entered in step 4 of each regression.

## Results

Primiparas comprised 61.1% of the sample (*n *= 157). Delivery modes were 73.9% vaginal (*n *= 190) and 26.1% caesarean (*n *= 67). Of the caesareans, 35 were planned (primipara = 17) and 32 were unplanned (primipara = 26). Correlations among variables of interest are reported in Tables A2 and A3. Maternal age and household income both correlated significantly with variables of interest so were also included as controls. Independent samples *t*-tests showed no significant demographic differences between mothers who did and did not complete the survey. There were no significant demographic differences between mothers who delivered vaginally and by caesarean.

The first moderated regression used overall subjective birth experience as the outcome variable (see Table A4). Household income, infant age, maternal age, parity and delivery mode did not relate to subjective birth experience. When RAQ dimensions were added in step 3, *R*^2^ increased from .03 to .18 (*F* change = 4.66, *p* < .001), explaining 18% of the variance in subjective birth experience. Perceived availability and feared loss of attachment figure both related to subjective birth experience and angry withdrawal was also related once interactions were included. The only significant interaction was feared loss × multiparous, indicating that feared loss was a more relevant attachment dimension for primiparas than multiparas.

The second moderated regression examined accuracy of expectations (see Table A5). Parity related to accuracy of expectations, with multiparas reporting more accurate expectations than primiparas. Separation protest related to accuracy of expectations, with higher accuracy being related to lower separation protest. Angry withdrawal also related to accuracy of expectations once interactions were included in the model, with more accurate expectations relating to higher reported angry withdrawal. Feared loss × multiparous was the only interaction that emerged, again being more salient for primiparas than multiparas.

The third moderated regression examined perceived control during labour and delivery (see Table A6). Household income and maternal age both related to perceived control, with higher income and younger maternal age relating to higher perceived control. Perceived availability was the only attachment dimension that related to perceived control. Feared loss × multiparous was again the only significant interaction that emerged, with feared loss being more related to perceived control for primiparas than multiparas.

The fourth moderated regression examined social support (see Table A7). The RAQ dimensions that were added in step 3 and increased the *R*^2^ from .01 to .23 (*F* change = 7.64, *p* < .001), indicating that 23% of the variance in social support could be attributed to attachment. Perceived availability, feared loss and compulsive care-seeking all significantly related to social support. Mode of delivery interacted with perceived availability and was more prominent for women who delivered vaginally. Mode of delivery also interacted with separation protest, which was more salient for mothers who delivered by caesarean.

The last moderated regression examined overall birth satisfaction (see Table A8). Women who delivered vaginally reported lower satisfaction than those who had caesareans, although this relation was no longer significant once attachment was included in the model. Feared loss was associated with satisfaction, with higher satisfaction relating to lower feared loss. Feared loss also interacted with parity, being more related to satisfaction for primiparas than multiparas. Parity also interacted with separation protest and was more related to satisfaction for multiparas than primiparas.

## Discussion

This study explored the relations between adult attachment and subjective birth experience, examining interactions between attachment, parity and mode of delivery. Mothers’ adult attachment was significantly related to subjective birth experience, and there were interactions between attachment, parity and mode of delivery. Further, different interactions emerged related to different aspects of birth experience, based on parity and mode of delivery.

Perceived availability, separation protest and feared loss of attachment figure were the attachment dimensions that stood out as being consistently related to aspects of subjective birth experience. These dimensions are especially meaningful because they tap into the secure base and safe haven aspects of attachment – protesting separations and confidence that your attachment figure will be present and accessible. These results suggest that birthing women need both a secure base from which to engage in this stressful experience and a safe haven for retreat when the pain and momentousness of the task becomes overwhelming.

Feared loss of attachment figure emerged as more salient for primiparas than multiparas. Primiparas have never given birth before and their lack of experience may result in fears about what is happening to them and the amount of control they have during childbirth. They are also becoming parents for the first time and may be anxious or frightened about changes in their adult attachment relationship in light of the family transition. Having a child for the first time is meaningfully different from having a second child (Kuo, Volling, & Gonzalez, 2017; Volling, 2012; Volling, Oh, Gonzalez, Kuo, & Yu, 2015). Both experiences include additions to the family, but first-time parents experience a new transition in their own relationship dynamic as well (Cox, Paley, Burchinal, & Payne, 1999; Lawrence, Cobb, Rothman, Rothman, & Bradbury, 2009), as they are no longer only attachment figures for one another, but also now co-parents and attachment figures together for their baby. Thus, the results suggest that perceived availability and feared loss might hold different meanings for primiparas and multiparas.

Angry withdrawal and compulsive care-seeking were the two insecure attachment dimensions that were related to different aspects of subjective birth experience. Women who were high in angry withdrawal reported worse overall birth experiences yet more accurate expectations than other mothers. These findings could be because of both the difficulty and nature of the physical experience. Labour is painful and distressing, but the birthing process cannot end until the baby is born; therefore, mothers who tend to withdraw angrily when distressed or in need cannot do so. This pattern suggests that these mothers may need particular support in planning their birth and developing strategies for coping with labour rather than withdrawing. Mothers high in compulsive care-seeking reported worse experiences of social support during labour and delivery than other mothers. These mothers desire higher levels of closeness and support and often feel that the support they receive is not adequate. This finding suggests that these mothers may be in need of greater social support during labour and delivery than other mothers. Together, these results indicate that mothers’ typical strategies for dealing with distress, particularly insecure strategies, should be taken into account when considering their support needs during childbirth.

Mode of delivery, surprisingly, did not relate to overall subjective birth experience. Closer examination, however, found an interaction between mode of delivery and attachment for social support during labour and delivery. Perceived availability of attachment figure was more salient in the experiences of social support for women who delivered vaginally than by caesarean. Womens’ attachment figures are likely to serve as their birthing partner and coach, helping them prepare and providing support through the process. The perception that partners are available to them during labour and delivery may thus be more relevant for those who give birth vaginally than by caesarean. A caesarean section is major surgery and therefore necessitates more professional intervention than a vaginal delivery, leading to the possibility of separation from the mothers’ support figures. Indeed, separation protest was more salient for mothers who delivered by caesarean. Caesarean delivery holds the possibility that mothers may be isolated from their attachment figures during the baby’s birth. Isolation from attachment figures can intensify distress, as can other cues to danger experienced in an operating room (Bowlby, 1973), all of which could prime mothers’ fears about separation from their attachment figures. Even if they are not separated, mothers undergoing a caesarean may feel emotionally isolated if they cannot receive the support that they need or expect from their attachment figures. Mothers’ experiences of social support influence their birth experiences, and this influence can persist years after birth (Sigurdardottir et al., 2017). These results provide further evidence that while mothers’ social support needs may vary with mode of delivery (DiMatteo et al., 1996), it is clearly important for all birthing women to have access to their secure base and safe haven during childbirth.

## Limitations and future directions

Sample size prevented examination of delivery mode more specifically than comparing vaginal and caesarean, and whether multiparas used the same delivery method as their previous birth(s). Subjective birth experiences following planned versus emergency caesareans differ (Lobel & DeLuca, 2007) and assisted vaginal deliveries have been associated with higher levels of distress than any other delivery mode (Maclean et al., 2000). Future research should examine birth experiences across modes of delivery as precisely as possible.

This sample was predominantly white, middle- to upper-middle class, and educated mothers living in the USA. While this study controlled for maternal age and household income, future research should examine these processes in more diverse samples. Subjective birth experiences, such as satisfaction and experience of social support, can differ between cultures, even within Western cultures (Burduli, Barbosa-Leiker, Fleming, Hollins Martin, & Martin, 2017; Tarkka, Paunonen, & Laippala, 2000). Cross-cultural examinations of attachment and birth experience should be a goal for future research.

Mothers self-reported all data retrospectively at one time point during the first year after giving birth. This cross-sectional approach means that any changes in mothers’ primary attachment relationship(s) since delivery or changes in mothers’ memories of their birth experiences cannot be accounted for in our analyses, although we did control for time since delivery. The self-report nature of the data also limits the generalisability of these results to actual maternal behaviour. Additionally, it would be ideal for data to be collected from fathers as well. Birth is an event that usually includes the entire family and examinations of fathers’ experiences of birth to date indicate that their experiences are meaningfully different from mothers (Hasman, Kjaergaard, & Esbensen, 2014; Nystedt & Hildingsson, 2017; Skari et al., 2002; Wilson et al., 2007). This is particularly relevant in an attachment framework if the father is the mother’s primary attachment figure and there are potential changes in their attachment relationship following the arrival of a new baby (e.g. Reisz, 2016). A prospective design where attachment is assessed observationally during pregnancy for both parents and examined as a predictor of subjective birth experience would be preferable. Future studies could extend this to assess how attachment and birth experience interact to predict parents’ care-giving representations and behaviours.

## Conclusion

Adult attachment and birth experience is a small but promising area within the broader empirical exploration of reproductive psychology and this study adds to the growing literature on attachment and birth experience. Findings from the present study demonstrated that the safe haven and secure base functions of adult attachment are meaningful for the subjective experience of women giving birth. For practitioners working with birthing and new mothers, attachment should be considered an important factor and support from her attachment figure should be facilitated wherever possible. Future research will hopefully continue to expand our understanding of the complex connections between mothers’ attachment and their subjective birth experiences.

## Appendix A

**Table A1.** Descriptive frequencies.

		Frequency	Percent
Parity	Primipara	157	61.1
	Multipara	100	38.9
Delivery mode	Vaginal	190	73.9
	Caesarean	67	26.1
Education	Some high school	5	1.9
	High school degree/GED	15	5.8
	Some college	60	23.3
	College degree	81	31.5
	Some graduate school	12	4.7
	Graduate degree	84	32.7
Household income	Under $10,000	10	3.9
	$10,000–$25,000	23	8.9
	$25,000–$50,000	53	20.6
	$50,000–$75,000	47	18.3
	$75,000–$100,000	43	16.7
	Over $100,000	81	31.5
Ethnicity	Asian	12	4.7
	Black	7	2.7
	Latina	15	5.8
	Mixed	21	8.2
	White	202	78.6
Marital status	Married/cohabiting	245	95.3
	Separated/divorced with visitation	4	1.6
	Single, no other parent	8	3.1

**Table A2.** Spearman’s rho correlations between subjective birth experience and attachment goal-related dimensions.

	1	2	3	4	5	6	7	8	9	10	11	12	13	14
1. Maternal age	1													
2. Baby age	–12	1												
3. Household income	.29***	–.21**	1											
4. Multiparous	.27	–.05	.07	1										
5. Caesarean	.078	.05	.15*	–.03	1									
6. Accuracy of expectations	.05	–.10	–.04	.15*	–.08	1								
7. Perceived control	–.10	–.06	.10	.08	–.12	.45***	1							
8. Social support	–.03	–.02	–.08	–.04	–.04	.30***	.34***	1						
9. Satisfaction with birth	–.04	–.10	.03	.08	–.14*	.66***	.59***	.44***	1					
10. Perceived availability of AF	.00	.14*	–.00	–.08	.11	–.28***	–.20**	–.38***	–.34***	1				
11. Feared loss of AF	–.02	.11	–.10	–.10	.06	–.22***	–.19**	–.32***	–.28***	.60***	1			
12. Proximity-seeking	–.07	–.04	–.11	.02	.03	–.10	–.10	–.15*	–.05	.05	.20**	1		
13. Separation protest	–.14*	.00	–.15*	.02	–.04	–.19**	–.12*	–.14*	–.13*	.30***	.32***	.32***	1	
14. Use of AF	–.02	.31	–.00	.01	.06	–.13*	–.20**	–.20**	–.18**	.59***	.40***	.08	.17**	1

**p < *.05, ***p <* .01, ****p* < .001, Bonferroni corrected significance levels are underlined < .003.

AF, attachment figure.

**Table A3.** Spearman’s rho correlations between subjective birth experience and attachment-based insecure personality cluster dimensions.

	1	2	3	4	5	6	7	8	9	10	11	12	13
1. Maternal age	1												
2. Baby age	–12	1											
3. Household income	.29***	–.21**	1										
4. Multiparous	.27	–.05	.07	1									
5. Caesarean	.078	.05	.15*	–.03	1								
6. Accuracy of expectations	.05	–.10	–.04	.15*	–.08	1							
7. Perceived control	–.10	–.06	.10	.08	–.12	.45***	1						
8. Social support	–.03	–.02	–.08	–.04	–.04	.30***	.34***	1					
9. Satisfaction with birth	–.04	–.10	.03	.08	–.14*	.66***	.59***	.44***	1				
10. Angry withdrawal	–.04	–.09	–.08	–.04	.06	–.17**	–.19**	–.27***	–.24***	1			
11. Compulsive care-giving	–.13*	.08	–.07	.03	–.11	.05	.05	.06	.06	–.08	1		
12. Compulsive self-reliance	.02	.18**	–.02	–.03	.14*	–.19**	–.16*	–.25***	–.23***	.66***	.28***	1	
13. Compulsive care-seeking	–.11	.04	–.08	–.04	.06	–.11	–.07	–.15*	–.07	.32***	.24***	.19**	1

**p <* .05, ***p <* .01, ****p* < .001, Bonferroni corrected significance levels are underlined < .003.

AF, attachment figure.

**Table A4.** Hierarchical regression and interaction analyses for adult attachment dimensions, parity, and mode of delivery to overall subjective birth experience (*N* = 257).

	Model 1		Model 2		Model 3		Model 4	
Variable	*β*	*p*	*β*	*p*	*β*	*p*	*β*	*p*
Household income	.04	.59	.05	.47	.02	.75	.05	.48
Baby age	–.10	.12	–.09	.17	–.08	.21	–.08	.23
Maternal age	.01	.91	.01	.89	.01	.89	–.00	.98
Multiparous			.07	.24	.06	.34	.05	.37
Caesarean			–.12	.06	–.09	.15	–.07	.24
Perceived availability					–.31**	.00	–.30**	.01
Feared loss of AF					–.21*	.02	–.23*	.01
Proximity-seeking					–.10	.20	–.08	.29
Separation protest					–.07	.33	–.09	.19
Use of AF					–.01	.91	–.02	.88
Angry withdrawal					.19	.10	.27*	.04
Compulsive care-giving					.05	.44	.04	.57
Compulsive care-seeking					–.05	.54	–.06	.45
Compulsive self-reliance					.02	.85	–.03	.79
Multiparous × Perceived Availability							.14	.22
Multiparous × Feared loss of AF							–.23*	.016
Multiparous × Proximity seeking							–.14	.08
Multiparous × Separation protest							.12	.08
Multiparous × Use of AF							.00	.97
Multiparous × Angry withdrawal							.00	.99
Multiparous × Compulsive care-giving							–.06	.41
Multiparous × Compulsive care-seeking							.07	.36
Multiparous × Compulsive self-reliance							–.00	.97
Caesarean × Perceived availability							–.06	.63
Caesarean × Feared Loss of AF							–.11	.20
Caesarean × Proximity seeking							–.02	.78
Caesarean × Separation protest							.06	.38
Caesarean × Use of AF							.05	.65
Caesarean × Angry withdrawal							–.07	.57
Caesarean × Compulsive care-giving							.04	.58
Caesarean × Compulsive care-seeking							–.00	.98
Caesarean × Compulsive self-reliance							.08	.49
*R*^2^	.01		.03		.18		.24	
*F* for change in *R*^2^			2.61		4.66***		1.05	

**p *< .05, ***p *< .01, ****p* < .001.

AF, attachment figure.

**Table A5.** Hierarchical regression and interaction analyses for adult attachment dimensions, parity and mode of delivery to accuracy of expectations.

	Model 1		Model 2		Model 3		Model 4	
Variable	*β*	*p*	*β*	*p*	*β*	*p*	*β*	*p*
Household income	–.03	.61	–.03	.67	–.05	.44	–.02	.76
Baby age	–.12	.05	–.11	.08	–.11	.10	–.10	.11
Maternal Age	.05	.41	.05	.43	.05	.49	.04	.56
Multiparous			.13*	.04	.13*	.04	.13*	.04
Caesarean			–.08	.19	–.06	.35	–.05	.41
Perceived availability					–.13	.26	–.12	.30
Feared loss of AF					–.07	.41	–.13	.18
Proximity-seeking					–.09	.25	–.07	.38
Separation protest					–.14*	.05	–.18*	.02
Use of AF					–.06	.58	–.06	.61
Angry withdrawal					.21	.08	.29*	.03
Compulsive care-giving					.04	.59	.01	.95
Compulsive care-seeking					–.03	.69	–.03	.71
Compulsive self-reliance					–.09	.45	–.13	.28
Multiparous × Perceived availability							.20	.08
Multiparous × Feared loss of AF							–.21*	.03
Multiparous × Proximity-seeking							–.16	.06
Multiparous × Separation protest							.11	.13
Multiparous × Use of AF							.01	.95
Multiparous × Angry withdrawal							.06	.68
Multiparous × Compulsive care-giving							.01	.93
Multiparous× Compulsive care-seeking							.06	.49
Multiparous × Compulsive self-reliance							–.06	.63
Caesarean × Perceived availability							–.01	.91
Caesarean × Feared loss of AF							.01	.89
Caesarean × Proximity seeking							–.03	.76
Caesarean × Separation protest							.05	.49
Caesarean × Use of AF							.01	.89
Caesarean × Angry withdrawal							–.15	.23
Caesarean × Compulsive care-giving							–.03	.74
Caesarean × Compulsive care-seeking							–.02	.87
Caesarean × Compulsive self-reliance							.01	.96
*R*^2^	.02		.04		.12		.19	
*F* for change in *R*^2^			3.06*		2.02*		1.18	

**p *< .05, ***p *< .01, ****p* < .001.

AF, attachment figure.

**Table A6.** Hierarchical regression and interaction analyses for adult attachment dimensions, parity and mode of delivery to perceived control during labour and delivery.

	Model 1		Model 2		Model 3		Model 4	
Variable	*β*	*p*	*β*	*p*	*β*	*p*	*β*	*p*
Household income	.16*	.01	.17**	.01	.16*	.02	.16*	.02
Baby age	–.05	.48	–.04	.58	–.04	.55	–.04	.57
Maternal age	–.13	.05	–.13*	.05	–.14*	.03	–.15*	.03
Multiparous			.05	.38	.05	.41	.04	.48
Caesarean			–.09	.17	–.07	.30	–.07	.25
Perceived availability					–.25*	.02	–.25*	.03
Feared loss of AF					–.06	.50	–.05	.58
Proximity-seeking					–.11	.16	–.12	.15
Separation protest					–.06	.38	–.08	.27
Use of AF					–.03	.78	–.07	.53
Angry withdrawal					.06	.61	.11	.41
Compulsive care-giving					.04	.61	.03	.72
Compulsive care-seeking					–.01	.40	–.01	.92
Compulsive self-reliance					.10	.37	.09	.48
Multiparous × Perceived availability							.03	.77
Multiparous × Feared loss of AF							–.23*	.02
Multiparous × Proximity-seeking							–.14	.09
Multiparous × Separation protest							.12	.11
Multiparous × Use of AF							.03	.79
Multiparous × Angry withdrawal							–.03	.83
Multiparous × Compulsive care-giving							–.03	.64
Multiparous × Compulsive care-seeking							.13	.12
Multiparous × Compulsive self-reliance							.11	.36
Caesarean × Perceived availability							.12	.33
Caesarean × Feared loss of AF							–.16	.07
Caesarean × Proximity-seeking							.04	.66
Caesarean × Separation protest							–.00	.96
Caesarean × Use of AF							.14	.17
Caesarean × Angry withdrawal							–.12	.34
Caesarean × Compulsive care-giving							.04	.63
Caesarean × Compulsive care-seeking							.07	.47
Caesarean × Compulsive self-reliance							.03	.81
*R*^2^	.04		.05		.11		.20	
*F* for change in *R*^2^			1.39		2.02*		1.29	

**p *< .05, ***p *< .01, ****p* < .001.

AF, attachment figure.

**Table A7.** Hierarchical regression and interaction analyses for adult attachment dimensions, parity and mode of delivery to social support during labour and delivery.

	Model 1		Model 2		Model 3		Model 4	
Variable	*β*	*p*	*β*	*p*	*β*	*p*	*β*	*p*
Household income	–.01	.88	.00	.99	–.02	.71	–.02	.71
Baby age	–.02	.80	–.01	.84	.01	.92	–.01	.90
Maternal age	.07	.29	.08	.25	.08	.20	.07	.28
Multiparous			–.05	.48	–.08	.17	–.09	.13
Caesarean			–.06	.33	–.02	.71	.01	.91
Perceived availability					–.42***	.00	–.39***	.00
Feared loss of AF					–.27**	.00	–.29**	.00
Proximity-seeking					–.04	.57	–.02	.80
Separation protest					.09	.18	.09	.19
Use of AF					.08	.40	.06	.52
Angry withdrawal					.13	.24	.15	.22
Compulsive care-giving					.05	.42	.08	.26
Compulsive care-seeking					–.17*	.02	–.19*	.01
Compulsive self-reliance					.11	.30	.07	.50
Multiparous × Perceived availability							.01	.89
Multiparous × Feared loss of AF							–.03	.78
Multiparous × Proximity-seeking							–.02	.83
Multiparous × Separation protest							–.01	.85
Multiparous × Use of AF							–.03	.78
Multiparous × Angry withdrawal							–.01	.92
Multiparous × Compulsive care-giving							–.07	.27
Multiparous × Compulsive care-seeking							–.11	.14
Multiparous × Compulsive self-reliance							–.01	.96
Caesarean × Perceived availability							–.24*	.03
Caesarean × Feared loss of AF							–.15	.07
Caesarean × Proximity-seeking							–.07	.36
Caesarean × Separation protest							.19**	.01
Caesarean × Use of AF							.08	.39
Caesarean × Angry withdrawal							.15	.20
Caesarean × Compulsive care-giving							.10	.17
Caesarean × Compulsive care-seeking							–.03	.71
Caesarean × Compulsive self-reliance							.13	.22
*R*^2^	.01		.01		.23		.32	
*F* for change in *R*^2^			.70		7.64***		1.59	

**p *< .05, ***p *< .01, ****p* < .001.

AF, attachment figure.

**Table A8.** Hierarchical regression and interaction analyses for adult attachment dimensions, parity and mode of delivery to satisfaction with overall birth.

	Model 1		Model 2		Model 3		Model 4	
Variable	*β*	*p*	*β*	*p*	*β*	*p*	*β*	*p*
Household income	.02	.78	.04	.59	.01	.87	.06	.41
Baby age	–.10	.13	–.09	.18	–.08	.23	–.06	.34
maternal age	.01	.91	.01	.86	.03	.69	.02	.80
Multiparous			.05	.42	.03	.57	.03	.60
Caesarean			–.14*	.03	–.12	.06	–.10	.12
Perceived availability					–.21	.05	–.22*	.05
Feared Loss of AF					–.26**	.00	–.26**	.01
Proximity seeking					–.05	.50	–.05	.55
Separation protest					–.04	.58	–.06	.43
Use of AF					–.00	.99	.03	.76
Angry withdrawal					.15	.22	.22	.10
Compulsive care-giving					.04	.60	.03	.64
Compulsive cares-eeking					.04	.58	.02	.84
Compulsive self-reliance					–.00	.98	–.06	.60
Multiparous × Perceived availability							.12	.29
Multiparous × Feared loss of AF							–.21*	.03
Multiparous × Proximity-seeking							–.10	.24
Multiparous × Separation protest							.15*	.04
Multiparous × Use of AF							.00	.99
Multiparous × Angry withdrawal							–.04	.77
Multiparous × Compulsive care-giving							–.10	.17
Multiparous × Compulsive care-seeking							.13	.10
Multiparous × Compulsive self-reliance							–.03	.82
Caesarean × Perceived availability							–.05	.64
Caesarean × Feared loss of AF							–.06	.50
Caesarean × Proximity-seeking							–.01	.90
Caesarean × Separation protest							–.03	.68
Caesarean × Use of AF							–.07	.51
Caesarean × Angry withdrawal							–.04	.75
Caesarean × Compulsive care-giving							.05	.51
Caesarean × Compulsive care-seeking							–.02	.85
Caesarean × Compulsive self-reliance							.10	.39
*R*^2^	.01		.03		.14		.21	
*F* for change in *R*^2^			2.84		3.43**		1.09	

**p *< .05, ***p *< .01, ****p* < .001.

AF, attachment figure.
